# A New Conceptualization of Human Visual Sensory-Memory

**DOI:** 10.3389/fpsyg.2016.00830

**Published:** 2016-06-09

**Authors:** Haluk Öğmen, Michael H. Herzog

**Affiliations:** ^1^Department of Electrical and Computer Engineering, University of HoustonHouston, TX, USA; ^2^Center for Neuro-Engineering and Cognitive Science, University of HoustonHouston, TX, USA; ^3^Laboratory of Psychophysics, Ecole Polytechnique Fédérale de Lausanne (EPFL)Lausanne, Switzerland

**Keywords:** sensory memory, iconic memory, modal model, non-retinotopic memory, non-retinotopic processes

## Abstract

Memory is an essential component of cognition and disorders of memory have significant individual and societal costs. The Atkinson–Shiffrin “modal model” forms the foundation of our understanding of human memory. It consists of three stores: Sensory Memory (SM), whose visual component is called iconic memory, Short-Term Memory (STM; also called working memory, WM), and Long-Term Memory (LTM). Since its inception, shortcomings of all three components of the modal model have been identified. While the theories of STM and LTM underwent significant modifications to address these shortcomings, models of the iconic memory remained largely unchanged: A high capacity but rapidly decaying store whose contents are encoded in retinotopic coordinates, i.e., according to how the stimulus is projected on the retina. The fundamental shortcoming of iconic memory models is that, because contents are encoded in retinotopic coordinates, the iconic memory cannot hold any useful information under normal viewing conditions when objects or the subject are in motion. Hence, half-century after its formulation, it remains an unresolved problem whether and how the first stage of the modal model serves any useful function and how subsequent stages of the modal model receive inputs from the environment. Here, we propose a new conceptualization of human visual sensory memory by introducing an additional component whose reference-frame consists of motion-grouping based coordinates rather than retinotopic coordinates. We review data supporting this new model and discuss how it offers solutions to the paradoxes of the traditional model of sensory memory.

## Introduction

### Modal model of human memory

**Figure 1:**
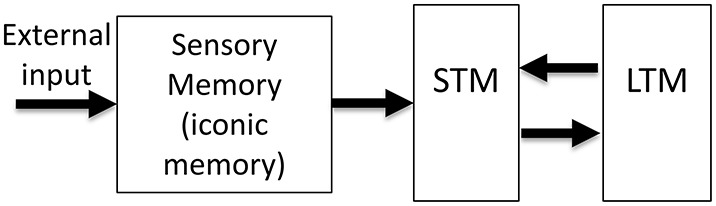
**The Atkinson-Shiffrin, or the “modal model” of human memory**. The external input is first stored in a large capacity but rapidly decaying store, called Sensory Memory (SM). Alternative terms for this stage include sensory register and sensory store. The visual component of SM is also called iconic memory. A distinction has been made between visible persistence vs. informational persistence in visual SM. Some authors use the term iconic memory only for the informational persistence component of visual SM, whereas others use for both visual and informational components. A subset of the contents of SM is transferred into a more durable (few seconds) but severely capacity-limited store, called Short-Term Memory (STM). Alternative terms for this stage include Short-Term Store, or Working Memory (WM). Some authors make a distinction between the use of the terms STM and WM. Finally, the contents of STM are transferred to Long-Term Memory (LTM), also called Long-Term Store, a store with very long duration and very large capacity.

The realization that human memory is not a unitary process but consists of multiple stores with distinct characteristics led to the Atkinson–Shiffrin, or the “modal” model of human memory (Atkinson and Shiffrin, 1968). As shown in Figure 1, this model consists of three major stores: The input is first stored in sensory memory (SM), which exhibits a very large capacity, but can maintain information only for a few hundred milliseconds. A subset of the contents of this rapidly decaying memory is transferred to Short-Term Memory (STM; also known as Working Memory WM). STM is severely limited in capacity and can hold information for several seconds to minutes. Finally, information is stored in Long-Term Memory (LTM), a store with very large capacity, capable of holding information as long as one's lifetime. Since its inception, the STM and LTM components of the modal model have undergone significant modifications (review: Baddeley, 2007), while SM has remained largely unchanged^1^.

### Sensory (iconic) memory

**Figure 2:**
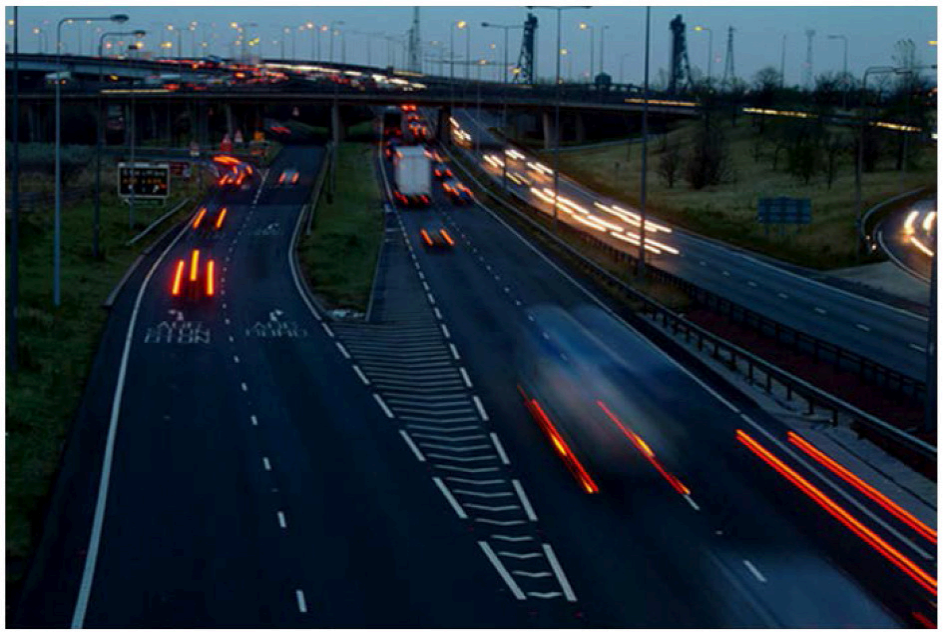
**A picture illustrating deleterious effects of a retinotopic Sensory Memory (rSM)**. As an object moves in the retinotopic space, its trace in rSM will create blur, as is the case for moving objects in the scene. Retinotopically shifted copies of the stimulus are superimposed and integrated, creating formless, ghost—like, appearances for moving objects. Similarly, the movements of observer's eyes, head, and body will induce retinotopic shifts in the image, blurring the whole scene and making the scene unrecognizable with a ghost-like appearance. Original photo from FreeFoto.com by permission.

The SM component of the modal model is based on Sperling's work in 1960s (Sperling, 1960; Averbach and Sperling, 1961). By using the partial-report technique, Sperling showed that a large-capacity visual memory stores information for few hundred milliseconds (Sperling, 1960) and more recent studies indicate that this information is not implicit and unconscious but rather directly reflects the phenomenal richness of our visual experience (Vandenbroucke et al., 2014). Early information processing theories viewed SM as a real-time buffer, which briefly stores the inputs impinging on the retina to allow attentional mechanisms to select a subset of this information for transfer to the limited capacity WM. However, subsequent analyses taking into account the properties of dynamic ecological viewing conditions showed that SM cannot fulfill this function during normal viewing conditions when objects or the subject are in motion; in fact, SM appears to be a hindrance to vision (Haber, 1983). A fundamental characteristic of iconic memory is that its contents are encoded in retinotopic coordinates (Haber, 1983; Irwin et al., 1983, 1988; Jonides et al., 1983; Rayner and Pollatsek, 1983; van der Heijden et al., 1986; Sun and Irwin, 1987). While a retinotopically encoded memory can serve a useful function when the observer and the objects in the environment are all static, it cannot store any meaningful information when the observer's eyes, head, body and external objects are in motion. Any relative motion between the observer's retinae and the external environment will cause a shift in retinotopic coordinates for the stimulus received by SM. These shifts, in turn, will cause blurring and inappropriate integration of information over space and time: A briefly presented stimulus remains visible for about 120 ms after its offset under normal viewing conditions (Coltheart, 1980), a phenomenon known as visible persistence (the visible component of SM^2^). Hence, if the input shifts in retinotopic coordinates, it will create partially processed copies of the stimulus that will be superimposed upon each other at different retinotopic locations, creating a blurred version of the stimulus. For example, given a visible persistence duration of 120 ms, an object moving at 8.3°/s will generate a blur trail of 1°. This motion blur is similar to pictures of moving objects taken by a camera at relatively slow shutter speeds mimicking the duration of visible persistence (Figure 2). Similarly, when the observer moves her head, body, and eyes, the retinotopic shift of stimuli engenders multiple blurred copies superimposed upon each other in SM. Since movements of the subject and the objects are characteristics of ecological normal viewing conditions, the emerging consensus has been that a retinotopically encoded memory cannot serve any useful function under normal viewing conditions. To explain our relatively sharp and clear percepts under normal viewing conditions, there have been several attempts to identify a spatiotopic version of this memory (Davidson et al., 1973; Ritter, 1976; White, 1976; Wolfe et al., 1978a,b; Breitmeyer et al., 1982; Jonides et al., 1982; McRae et al., 1987); however, these were unsuccessful (Haber, 1983; Irwin et al., 1983, 1988; Jonides et al., 1983; Rayner and Pollatsek, 1983; van der Heijden et al., 1986; Sun and Irwin, 1987) and this area of research has been stagnant for half-century.

To move forward, three fundamental questions need to be addressed:

Q1. How does the visual system process and store information non-retinotopically over space and time?
Q2. How does the visual system control deleterious effects of retinotopic sensory memory?
Q3. What purpose does a retinotopic sensory memory serve?

In the following we provide answers to these questions. First, to put retinotopy in the context of visual perception, in Section Metacontrast and Anorthoscopic Perception: A Retinotopically Extended Representation is Neither Sufficient nor Necessary for Vision we present evidence showing that retinotopic representations are neither sufficient nor necessary for visual perception. In Sections Sequential Metacontrast: Non-retinotopic Information Storage and Processing and The Ternus-Pikler Paradigm to Probe Retinotopic and Non-retinotopic Processes, we review briefly two experimental paradigms that we have used to demonstrate the existence of a non-retinotopic memory. Based on these findings, in Section A New Conceptualization of Human Sensory Memory, we present a modified version of the modal model with a new sensory memory component. In Section Paradoxes of Retinotopic Sensory Memory Revisited, we revisit the paradoxes associated with the retinotopic sensory memory and discuss how the new model offers resolutions to these paradoxes.

## Non-retinotopic information processing and storage

### Metacontrast and anorthoscopic perception: a retinotopically extended representation is neither sufficient nor necessary for vision

Assume that one briefly flashes a supra-threshold stimulus; the observer will clearly perceive the shape of this stimulus. Assume now that a second stimulus is flashed *after* this stimulus in a way that it surrounds but does not spatially overlap with the first stimulus. This second stimulus can render the first one completely invisible. This phenomenon is known as visual masking, which refers to the reduced visibility of one stimulus (target), due to the presence of another stimulus (mask) (Bachmann, 1994; Breitmeyer and Öğmen, 2000, 2006). Metacontrast is a specific type of visual masking, in which the target and mask do not overlap spatially. Hence in metacontrast, the target maintains its retinotopic representation, i.e., the mask does not reduce the visibility of the target by directly occluding the retinotopic representation of the target. Hence a retinotopic representation of a stimulus is *not sufficient* for its perception or storage. The mask may be interfering *indirectly* with the retinotopic representation of the mask; but why would the visual system suppress a perfectly visible stimulus when it is embedded in a dynamic context? In the following sections, we will re-visit visual masking and its role in controlling sensory memory in Sections Sequential Metacontrast: Non-retinotopic Information Storage and Processing and How the Visual System Controls Deleterious Effects of rSM: Motion Deblurring to answer the questions Q1 and Q2 above.

**Figure 3:**
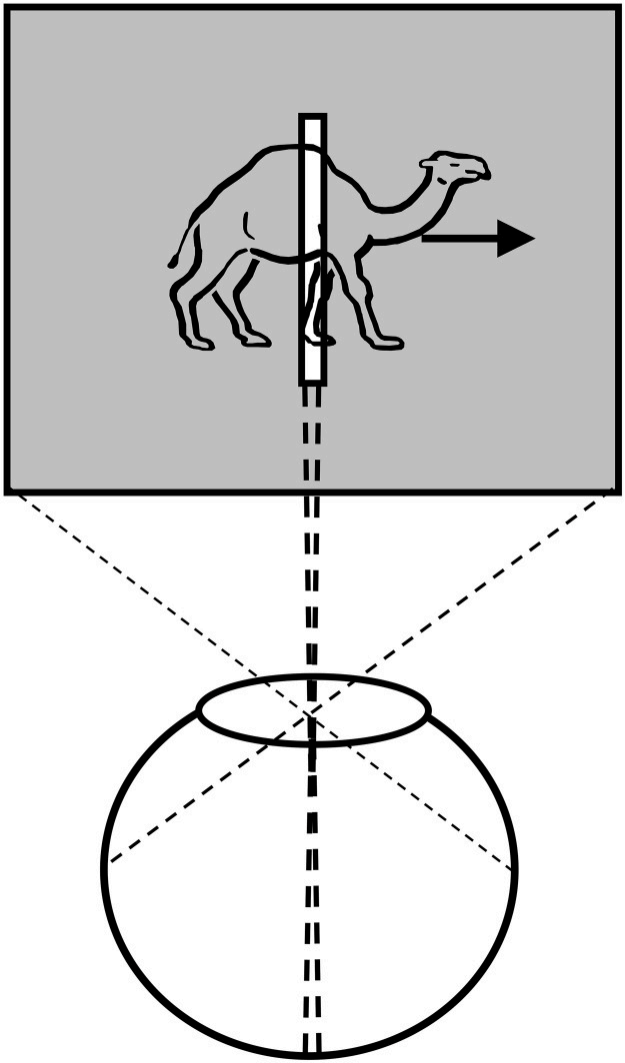
**Anorthoscopic perception**. A stimulus is moved behind an opaque region with only a very narrow opening in the form of a slit. As the object moves behind the slit, samples of its form taken along the slit fall unto the same narrow retinotopic region. Hence, these different narrow two-dimensional samples will be superimposed and integrated in the retinotopic sensory memory, making the perception of spatially extended form impossible. From Öğmen (2007).

Anorthoscopic perception, or slit viewing, is an experimental paradigm that derives its name from the anorthoscope, a device invented by Plateau in the nineteenth century (Plateau, 1836). Since its invention, variants of this device have been used to study human perception (e.g., Zöllner, 1862; Parks, 1965; Rock, 1981; Morgan et al., 1982; Sohmiya and Sohmiya, 1994; Öğmen, 2007; Aydin et al., 2008, 2009; Agaoglu et al., 2012). As depicted in Figure 3, a moving stimulus is viewed behind a narrow slit. All spatial information about the moving stimulus is restricted into a very narrow retinotopic strip. At any given time, only a very narrow spatial structure of the stimulus is visible. In other words, there is no spatially extended retinotopic representation for the moving stimulus. Moreover, as the stimulus moves, *different* parts of the object's shape fall onto the *same* retinotopic area. Hence, the contents of a retinotopic SM will consist of all stimulus parts mixed and blended into each other within the slit area. Yet, observers are able to spatiotemporally integrate the slit views to construct the spatially extended form of the moving stimulus in the absence of a retinotopically extended representation of the stimulus. Hence, anorthoscopic perception shows that a spatially extended retinotopic representation is *not necessary* for the perception of spatial form. Moreover, it also shows that, since information about different parts of the shape are shown at different time instants, the visual system is able to store this information and integrate it *non-retinotopically* in order to build the complete spatial layout of the stimulus. This indicates some type of non-retinotopic memory (Haber, 1983) but until recently it was not clear how it may be operating and its relation to the more traditional retinotopic SM. We will revisit anorthoscopic perception in Section Anorthoscopic Perception.

### Sequential metacontrast: non-retinotopic information storage and processing

**Figure 4:**
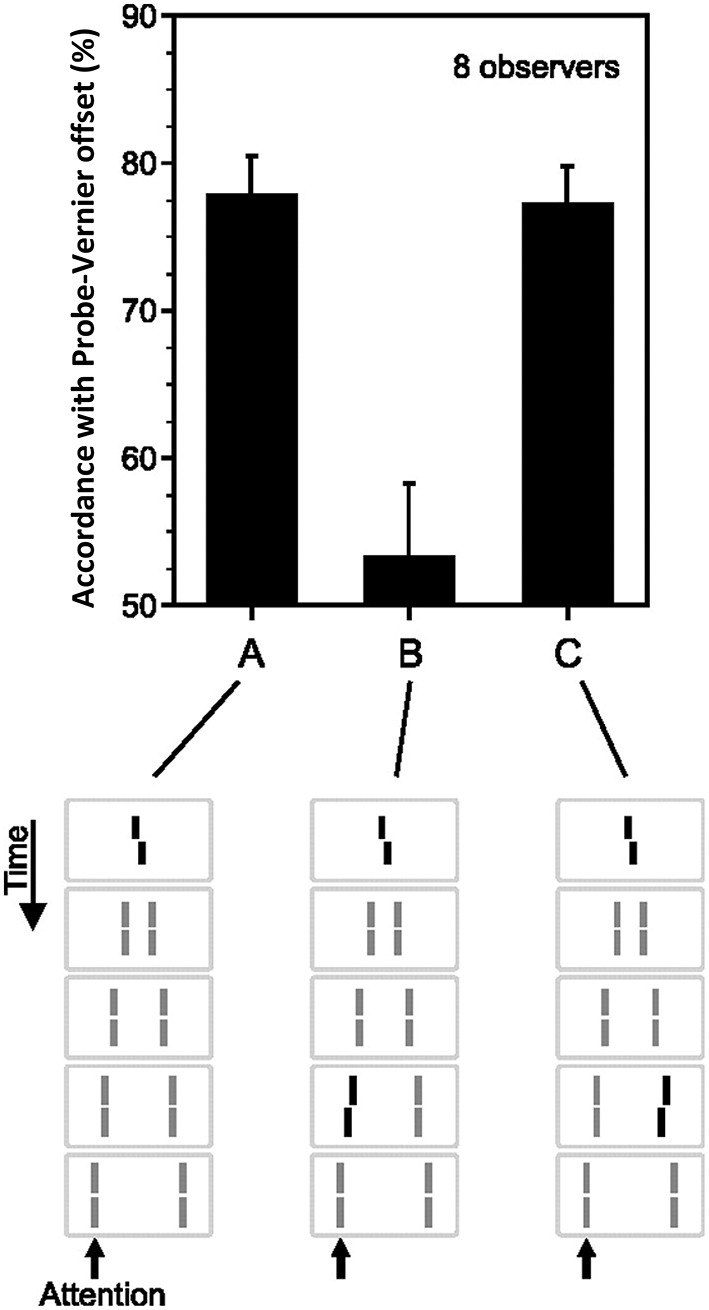
**Non-retinotopic memory revealed by sequential metacontrast**. The stimulus, shown at the bottom of the figure, consists of a central vernier, which is followed in time by a succession of spatially adjacent verniers. Observers perceive two motion streams, one to the left and one to the right, emerging from the center. A vernier offset, called the “probe-vernier,” is introduced to the central element. Observers report the perceived vernier offset (left or right) in the left motion stream (indicated by the arrow). In **(A)**, none of the flanking verniers has an offset. Observers' responses are in agreement with the offset of the probe-vernier in about 80% of the trials. In **(B)**, a vernier of opposite offset with respect to the probe vernier, called “anti-vernier,” is introduced to the left motion stream. Now, the agreement of the observers' response with the probe-vernier is near chance. This indicates that the information about the probe-vernier is stored in memory and is integrated with the anti-vernier so that the two cancel each other. The important point is that the storage and integration is *non-retinotopic,* since the probe-vernier and anti-vernier are presented at *different* retinotopic locations. In **(C)**, the anti-vernier is inserted to the rightward motion stream. In this case, the probe-vernier and anti-vernier are not integrated, showing that non-retinotopic storage and integration is specific to the motion stream. Adapted from Otto et al. (2006); the Association for Research in Vision and Ophthalmology (© ARVO).

Sequential metacontrast (Piéron, 1935; Otto et al., 2006) is a special case of metacontrast, consisting of *multiple* target and mask pairs as shown at the bottom of Figure 4. A central target is presented first, followed by two spatially flanking masks, which in turn are followed by lateral masks on one side, etc. Observers perceive two motion streams originating from the center, one to the left, and one to the right. With the appropriate choice of stimulus parameters, the central element can be completely masked making observers unable to tell whether it is presented or not (Otto et al., 2006). In order to test non-retinotopic storage and integration of information, we introduced a feature into this invisible central element in the form of a vernier offset (called “probe-vernier”), with a random spatial shift between its two segments, left or right, from trial to trial. Observers were asked to report the perceived vernier-offset in the left motion stream. Observers did not know if and where vernier offset(s) were presented in the display. In Figure 4A, all flanking lines are non- offset and the responses of the observers are above chance level with the offset of the probe-vernier. This indicates that the central *invisible* probe-vernier's offset direction is *stored in a non-retinotopic memory and attributed to the left motion stream,* a process that we call feature attribution. In Figure 4B, a vernier of opposite offset-direction is introduced into the left stream (in reference to the probe-vernier, this is called “anti-vernier” hereafter, because its offset direction is always opposite to that of the probe-vernier). Now, the agreement of observer's response with the offset direction of the probe-vernier is near 50%, i.e., the point of equal strength. This indicates that the two *verniers are integrated* in the non-retinotopic memory and as a result they cancel each other. Finally, in Figure 4C we show that this *integration is specific to the motion stream, i.e., the two verniers are integrated only if they belong to the same motion stream*. The probe-vernier is symmetric with respect to leftward and rightward motion streams; hence it will be attributed to both streams. The anti-vernier will be integrated with the probe vernier only in the specific motion stream where it appears. In Figures 4B,C, it will be integrated exclusively within the leftward and rightward motion streams, respectively. Since the observer is reporting the leftward motion stream, the integration is revealed in observer's response in Figure 4B but not in Figure 4C. Taken together, these results show that information presented at the central retinotopic location is stored in memory and is integrated with the information presented at other retinotopic locations according to the motion of stimuli, hence, providing evidence for a *non-retinotopic memory that depends on stimulus motion.* Additional results supporting this finding (with multiple vernier's inserted at multiple locations) can be found in Otto et al. (2006, 2009, 2010a,b).

A methodological difference between traditional studies of memory and the sequential metacontrast paradigm outlined above is the ways cues are used. In the partial-report technique, after the offset of the stimulus, a cue is delivered (with a delay) to indicate which item(s) to report (Sperling, 1960). As soon as the cue is delivered, the observer can initiate the reporting process. More recent studies combined change-detection paradigms with *retro*-cueing to investigate memory processes, in particular STM (e.g., Griffin and Nobre, 2003; Sligte et al., 2008; Hollingworth and Maxcey-Richard, 2013; Rerko et al., 2014; van Moorselaar et al., 2015). In these studies, an array of items is presented, followed by a retro-cue, and finally by a comparison item or display. The task of the observer is to report whether the cued item has changed. Hence, in this paradigm, the cue itself is not sufficient to initiate the response. Sperling's original purpose for introducing the cue was to design a *partial* report task so as to avoid the decay of information during the time it takes to report the contents of the memory. In addition to indexing the items to be reported, cues also guide attention and hence allow the examination of the role attention may play in the storage, maintenance, or transfer of information in memory. For example, a retro-cue indicates to the observer which particular memory item to attend in order to complete the impending comparison.

We have combined cueing with sequential metacontrast to examine the role of attention in non-retinotopic memory (Otto et al., 2010a). In a first experiment, we used a stimulus as shown in Figure 4. The stimulus could contain only a central vernier (as in Figure 4A), a central and a flanking vernier (as in Figures 4B,C), or only a flanking vernier. In the experiment described in Figure 4, the observers were instructed at the beginning of the block of trials which motion stream to attend (Otto et al., 2006). In Otto et al. (2010a), we used an auditory cue that indicated which motion stream (leftward or rightward) to attend. We varied the timing of the auditory cue with respect to the stimulus. When the cue was delivered before stimulus onset, observers focused their attention exclusively on the cued stream. By delaying the cue, we could control when unifocal attention could be devoted to a particular motion stream. Accordingly, the cue could focus attention preferentially on the central or the flanking vernier depending on its timing with respect to the onset of the central and flanking vernier. Our results showed that neither the timing nor the distribution of attention (focused on one stream vs. distributed to both streams) had any specific effect on non-retinotopic feature integration. These findings indicate that attention cannot directly access single lines and mandatory feature integration occurs within the attended motion stream.

### The ternus-pikler paradigm to probe retinotopic and non-retinotopic processes

**Figure 5:**
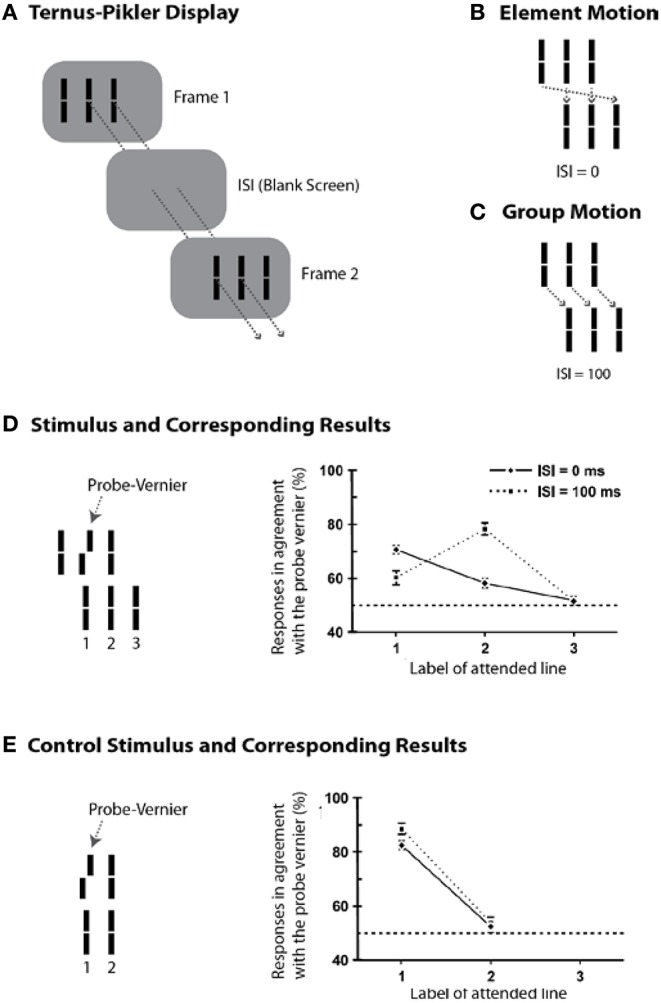
**The Ternus-Pikler paradigm to pit directly retinotopic processes against non-retinotopic processes. (A)** The Ternus-Pikler display consists of three elements shown in a first frame, followed by a blank screen, and finally another frame where the three elements are shifted by one inter-element distance so that two of the elements in the two stimulus frames fall exactly on the same retinotopic locations. When the duration of the blank screen, i.e., the Inter-Stimulus Interval (ISI) between the two stimulus frames is short, “element motion” is perceived: As depicted by the arrows in **(B)**, the retinotopically overlapping elements are perceived stationary whereas the leftmost element in the first frame is perceived to move to the rightmost element in the last frame. When ISI is long, “group motion” is perceived: As depicted by the arrows in **(C)**, the three elements are perceived to move as a group. **(D)** A probe-vernier is inserted to the central element in the first frame and observers reported the perceived vernier offset for elements marked 1, 2, and 3 in the second frame. In the retinotopic memory, the probe-vernier should be integrated to element 2, which shares the same retinotopic location, regardless of the value of ISI as long as it is within the integration time-window of SM. However, if memory is non-retinotopic and is based on a reference-frame that follows motion grouping, then the probe-vernier should be integrated to element 1 in the case of element motion and to element 2 in the case of group motion (see the motion correspondences in **B**,**C**). The results, shown on the right, support the predictions of motion-grouping based non-retinotopic memory. **(E)** In the control display, the flanking elements are removed and no motion is perceived. In this case, both retinotopic and non-retinotopic memories predict the same outcome, namely, the probe-vernier should be integrated to element 1 for both ISI values. This is indeed what the results show. Adapted by permission from Öğmen et al. (2006).

Whereas the sequential metacontrast paradigm provides evidence for non-retinotopic memory, it does not pit directly retinotopic and non-retinotopic processes against each other. In order to achieve this goal, we developed an experimental paradigm that can pit directly retinotopic and non-retinotopic memories against each other, while revealing the direct role of motion in the process. To this end, we modified a stimulus paradigm developed by Gestalt psychologists Ternus and Pikler (Pikler, 1917; Ternus, 1926). Figure 5A shows a basic Ternus-Pikler display. The first frame contains three elements. After an inter- stimulus interval (ISI), these three elements are shifted to the right by one inter-element distance so that two of the elements overlap retinotopically across the two frames (these retinotopically overlapping elements allow the testing of retinotopic information storage and integration). For small values of ISI, observers report seeing the leftmost element of the first frame move to the rightmost element of the second frame, while the other two elements appear stationary (Figure 5B). This percept is called “element motion” (Pantle and Picciano, 1976). For longer ISIs, observers report seeing all three elements moving in tandem to the right as a group (Figure 5C). This percept is called “group motion.” These motion-based non-retinotopic correspondences between the elements in the two frames allow the testing of motion-based, non-retinotopic information storage and integration. The probe-vernier was inserted to the central element of the first frame as shown in Figure 5D-left (Öğmen et al., 2006). We asked observers to report the perceived offset-direction for elements in the second frame, numbered 1, 2, and 3 in Figure 5D-left. None of these elements contained a vernier offset and naïve observers did not know where the probe-vernier was located. Consider first the control condition in Figure 5E, obtained by removing the flanking elements from the two frames. In this case no motion is perceived. Based on the traditional retinotopic iconic memory account, the information about the probe vernier should be stored at its retinotopic location and it should be integrated with element 1 in the second frame, which occupies the same retinotopic location. Thus, the agreement of observers' responses with the direction of probe-vernier offset should be high for element 1 and low for element 2. In agreement with the large body of findings on iconic memory, this is indeed what we found (data in Figure 5E-right). A retinotopic iconic memory predicts the same outcome for the Ternus-Pikler display regardless of whether element or group motion is perceived, as long as the ISI is within the time-scale of iconic memory. On the other hand, if there is a memory mechanism that stores and integrates information non-retinotopically according to motion grouping relations (Figures 5B,C), one would expect the probe vernier to integrate with element 1 in the case of element motion (Figure 5B) and with element 2 in the case of group motion (Figure 5C). Our results supported the predictions of the non-retinotopic motion- based memory hypothesis (5D-right). Additional results supporting this finding (with multiple vernier's inserted at multiple locations) can be found in Öğmen et al. (2006), Scharnowski et al. (2007), Otto et al. (2008), Boi et al. (2009, 2011), and Noory et al. (2015a,b).

## A new conceptualization of human sensory memory

**Figure 6:**
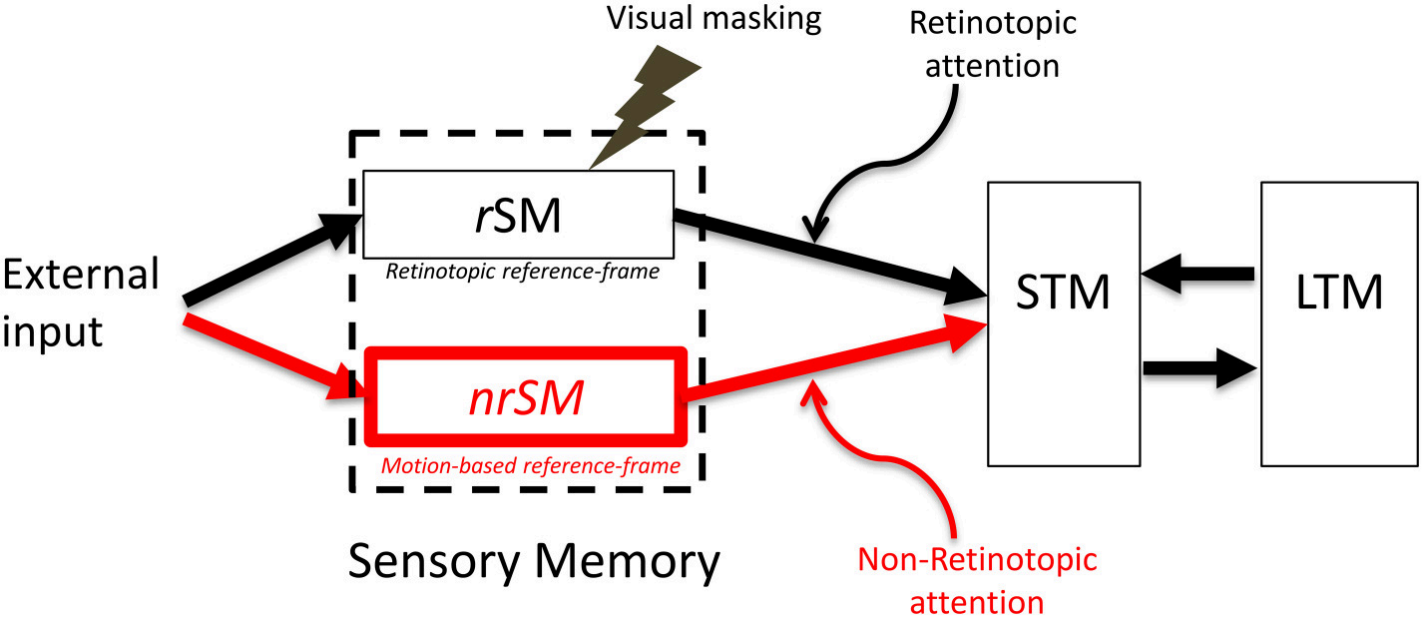
**The proposed new model**. The SM stage of the modal model is modified to include two stores, a retinotopic SM (*r*SM), corresponding to the SM in the modal model and an additional non-retinotopic SM (*nr*SM). The reference-frame for *r*SM is anchored in retinotopic coordinates, whereas the reference-frame of *nr*SM is non-retinotopic, flexible and based on motion groupings in the stimulus. Hence, *nr*SM can have simultaneously multiple reference-frames depending on how the stimulus generates motion groupings (see Figure 7, for an example). The contents of *r*SM can be suppressed by visual masking whereas *nr*SM is immune to masking. Information from *r*SM and *nr*SM is transferred to STM through retinotopic and non-retinotopic attention mechanisms.

Extensive research supports the existence of a retinotopic sensory memory (review: Coltheart, 1980). The research reviewed in the previous section supports the existence of a *non-retinotopic, motion-based,* sensory memory. Taken into account these recent findings, we modified the sensory memory component of the modal model by introducing a non-retinotopic store (Figure 6). To be consistent with the terminology used in the literature, we keep the term iconic memory for the retinotopic component of sensory memory and also refer to this component as the “*retinotopic Sensory Memory”* (*r*SM). We call the newly introduced non-retinotopic component, the “*non-retinotopic Sensory Memory”* (*nr*SM).

**Figure 7:**
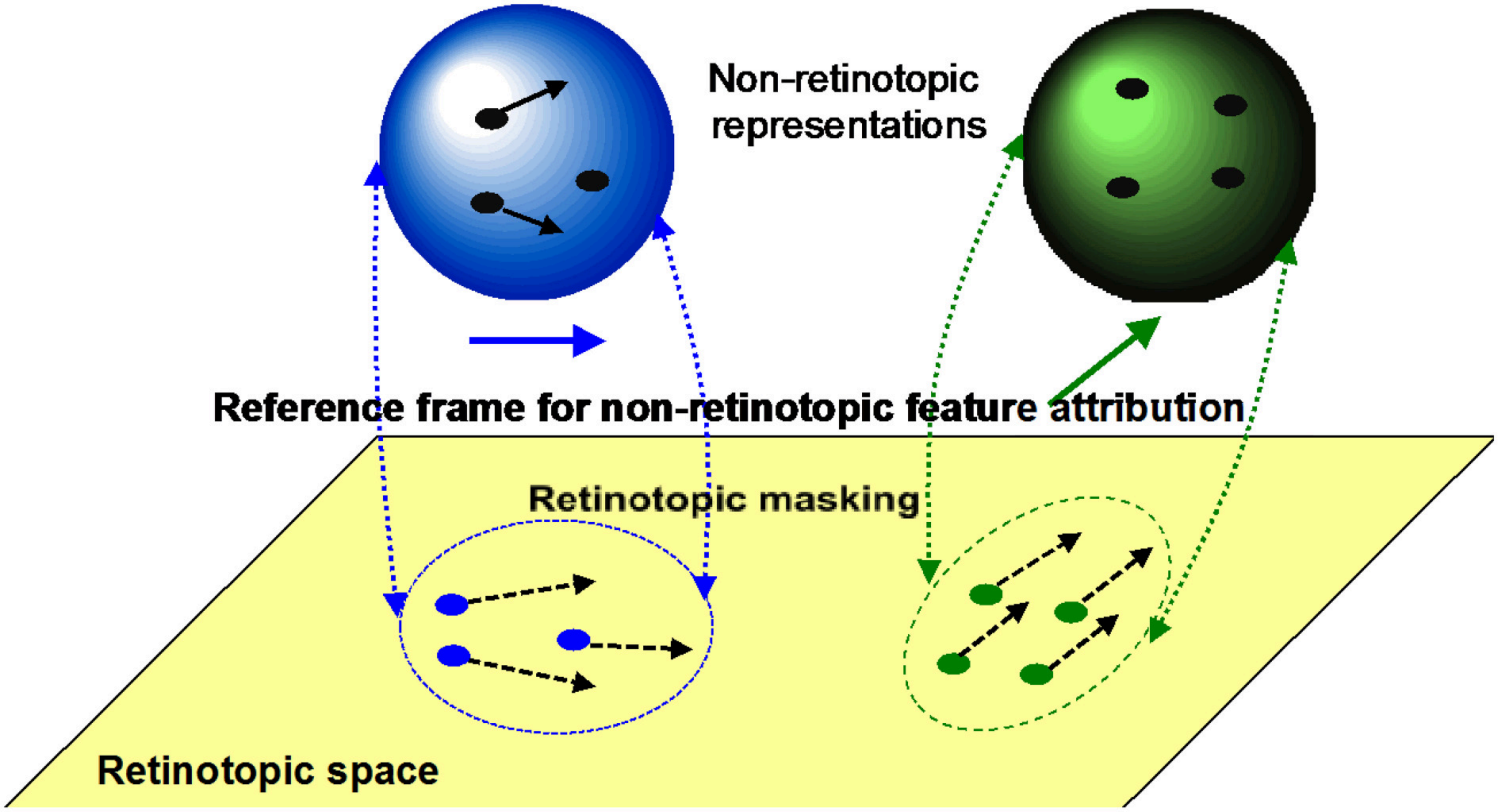
**The operation of *nr*SM**. First, a motion analysis is carried out in the retinotopic areas and stimuli are grouped according to Gestalt grouping principles (e.g., common fate). In this example, two groups are formed, the rightward moving blue dots, and upward moving green dots. For each group, a common motion is extracted as the reference-frame and stimuli are transferred into non-retinotopic representations according to this reference-frame. The non-retinotopic representations are depicted at the top of the figure. *nr*SM stores information within this non-retinotopic representation.

**Figure 8:**
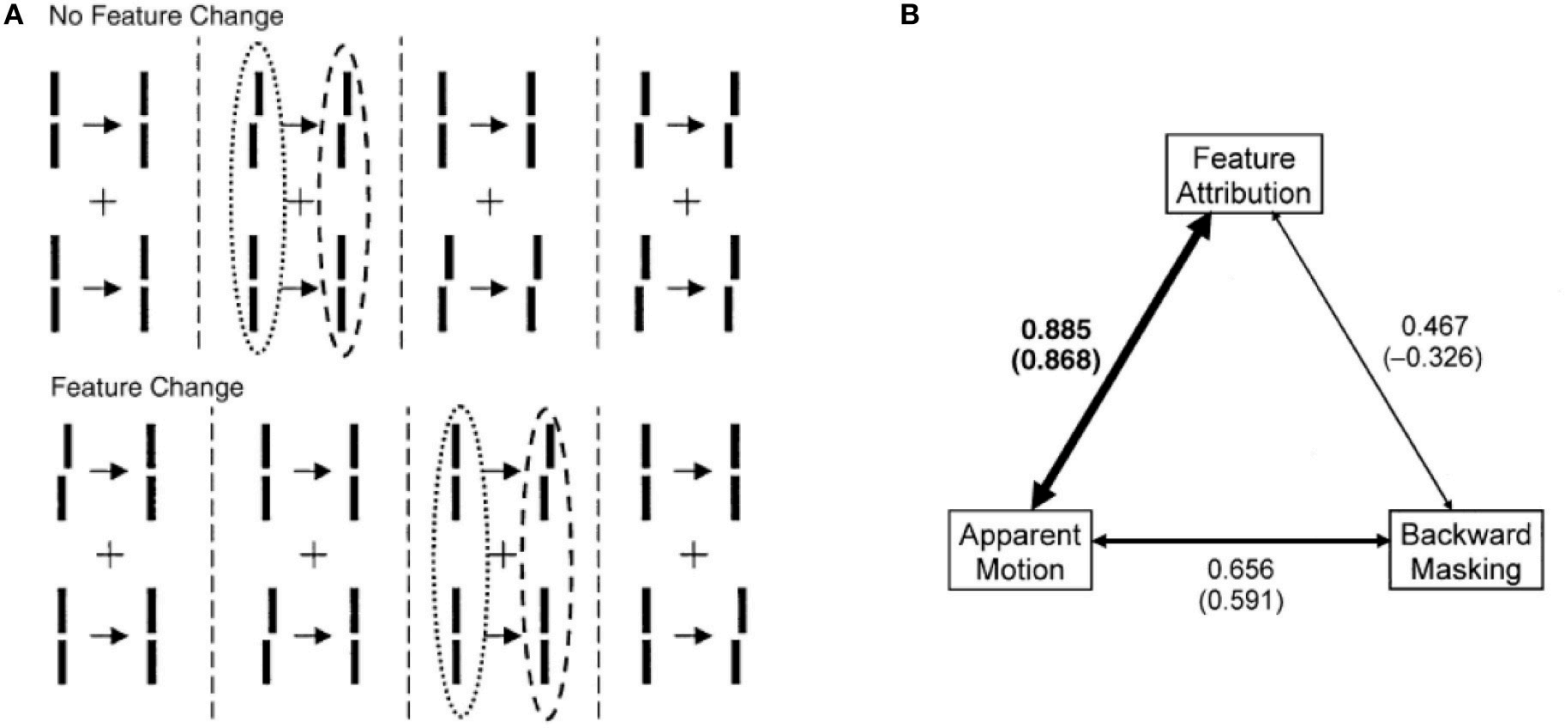
**Stimuli consisted of a first frame containing two verniers at the left of the fixation, followed after an ISI by a second frame where the two verniers were presented to the right of the fixation cross**. Vernier offsets were introduced so that either there was no change between the two frames, as illustrated by the examples at the top of **(A)**, or there was a change from the first to the second frame (bottom of **A**). In separate session, we asked observers (i) to rate the strength of smooth apparent motion (motion task), (ii) to report whether the upper and lower verniers in the first frame had the same offset or not (masking task), or (iii) to report whether the upper and lower verniers in the second frame had the same offset or not (feature attribution task). The feature attribution task reflects the operation of *nr*SM. **(B)** Correlations between motion, masking, and feature attribution. The width of the arrows is directly proportional to strength of the correlations. For each pair of variables, bivariate correlations are given without parentheses and partial correlations are given in parentheses. Only the correlation between feature attribution (*nr*SM) and motion was significantly larger than zero (indicated by boldface). From Breitmeyer et al. (2008).

**Figure 9:**
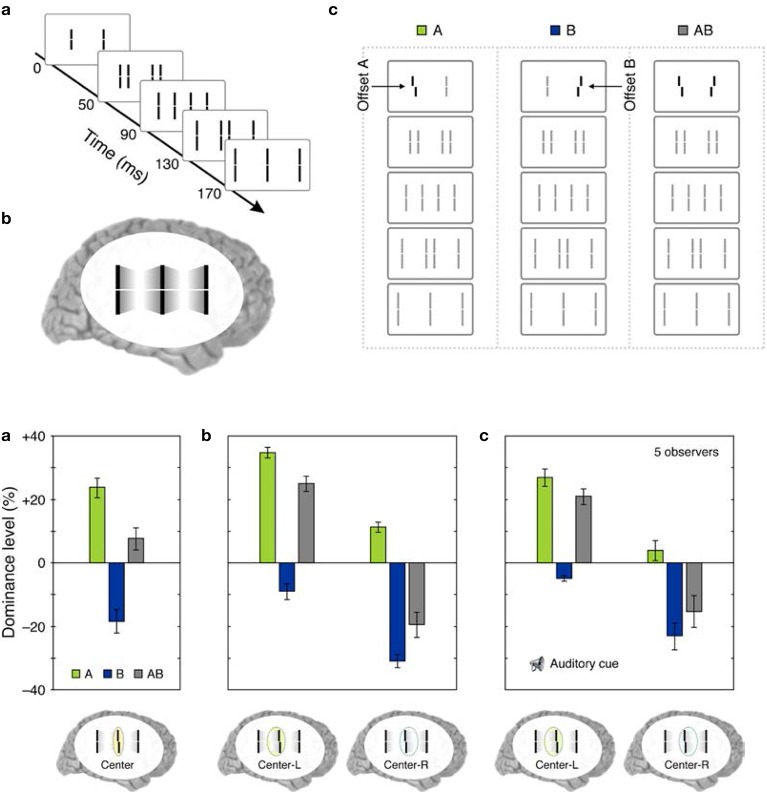
**Top panel: (a) The stimulus consisted of four motion streams emerging from two elements**. Two of these motion streams merged to a common element in the last frame. **(b)** The percept. **(c)** A probe-vernier was inserted to the left element in the first frame (A), an anti-vernier was inserted to the right element in the first frame (B), or both the vernier and the anti-vernier were present (AB). **Bottom panel: (a)** Observers were instructed to focus their attention on the common central element in the last frame and report the perceived vernier offset for this element. In conditions A and B, the probe-vernier and anti-vernier dominate responses, respectively, as indicated by positive and negative values of dominance level. In condition AB, the dominance level equals approximately to the sum of individual dominance levels of A and B. **(b)** Observers were instructed to report the perceived vernier offset of the central element in the last frame while focusing their attention either on the left (Center-L) or right (Center-R) motion streams. Focusing attention on the left frame increases the dominance of the probe-vernier and decreases the dominance of the anti-vernier. Focusing attention on the right stream has the opposite effect. **(c)** The same attentional modulation as in **(b)** is observed even when the stream to focus attention was indicated by an auditory cue is delivered with a cue-stimulus onset asynchrony of 500 ms, i.e., more than 300 ms after the two motion streams merged to the common element. Adapted from Otto et al., 2010a; the Association for Research in Vision and Ophthalmology (© ARVO).

Below, we discuss the fundamental properties of the new model and the key differences between its *r*SM and *nr*SM components. Specifically, we will discuss the differences in terms of reference-frames used in each, how masking affects the contents of each memory component, the distinct but complementary roles masking and motion play with respect to these two components, and finally the influence of attention mechanisms:

- *Retinotopic vs. motion-based reference-frames*: Whereas the reference-frame, or the coordinate system, of rSM is anchored in retinotopic coordinates, nrSM uses motion-grouping-based non-retinotopic reference-frames or coordinate systems. Figure 7 depicts the operations underlying nrSM. At a first stage, motion is analyzed within retinotopic representations and motion vectors are grouped according to Gestalt grouping principles (e.g., common fate). For example, in Figure 7, the moving dots are grouped into two distinct groups based on their direction of motion. For each group, a common motion vector is computed and this common motion vector serves as the reference-frame according to which the contents of memory are encoded. As illustrated in the example, multiple motion groupings can be extracted simultaneously across the visual field and hence nrSM can contain multiple reference-frames, unlike rSM which has a single reference-frame anchored in retinotopic coordinates.
- *Immunity to masking:* In the experiments discussed in Section Sequential Metacontrast: Non-retinotopic Information Storage and Processing, the probe-vernier can be completely masked; however, the information about its vernier-offset is not masked since it is integrated to other verniers in the motion stream and observers can reliably report the direction of vernier offset in behavioral experiments. By using the Ternus–Pikler display, we tested directly whether masking operates in retinotopic coordinates and whether nrSM is susceptible to masking (Noory et al., 2015b). Our results showed that masking operates in retinotopic coordinates and nrSM is immune to masking (Noory et al., 2015b). Hence, unlike rSM whose contents are suppressed by masking (Averbach and Coriell, 1961; Averbach and Sperling, 1961; Coltheart, 1980), the contents of nrSM are immune to masking Noory et al., 2015b
- *Distinct and complementary roles of masking and motion in sensory memory*: Masking “turns off” rSM whereas motion “activates” nrSM^3^. To test the proposed distinct but complementary roles of masking and motion, we determined the correlations between the non-retinotopic storage and integration in nrSM (we call this effect “feature attribution” since features are not perceived according to their retinotopic coordinates but are attributed non-retinotopically to motion streams), masking, and motion (Breitmeyer et al., 2008). The first frame contained a vernier pair presented to the left of the fixation cross. The second frame contained a vernier pair presented to the right of the fixation cross. Offsets were introduced so that features (vernier offset) either changed or remained the same across the two frames (see Figure 8). In the feature-attribution task, subjects judged the vernier pair presented in the second frame and reported whether the upper and lower verniers in the second frame were the same or different (examples highlighted by dashed ovals). For example, the correct response is “same” for the rightmost stimulus sequence in the top panel of Figure 8 and “different” for the rightmost stimulus sequence in the bottom panel. On trials in which feature attribution occurred, a larger number of misidentifications of the vernier pair presented in the second frame would be expected when the stimulus sequences had feature changes than when they did not. Hence, the difference between the numbers of misidentifications in the no-change and feature-change conditions provide an index of feature attribution, with larger differences corresponding to stronger feature attribution. Since feature attribution requires temporal storage and non-retinotopic integration, it measures rSM. In the backward-masking task, subjects judged the vernier pair in the first frame and reported whether the upper and lower verniers were the same or different (examples highlighted by dotted ovals). In the apparent motion task, observers rated the strength of smooth apparent motion, using a scale ranging from 0 (no motion) to 5 (optimal smooth motion). Figure 8B shows the correlations between these three variables computed across several values of stimulus-onset asynchronies between the two frames. As one can see from the figure, feature attribution correlated strongly with motion (significance: *p* < 0.01 for bivariate correlation and *p* < 0.03 for partial correlation) while the correlation of feature attribution with masking was weaker and not significant (*p* > 0.175 for bivariate correlation and *p* < 0.295 for partial correlation). Thus, these results support that the operation of *nr*SM has strong correlation with motion, which according to our theory constitutes its reference-frame, whereas the effect of masking is related to the operation of *r*SM.
- *Retinotopic vs. non-retinotopic attention mechanisms:* Attention is a key process that controls the transfer of information from SM to STM and various lines of evidence suggest that temporal dynamics of SM plays a fundamental role in determining how attention can select information from SM for transferring into STM (Wutz and Melcher, 2014). Attentional processes can be classified into two broad types, endogenous and exogenous (e.g., Posner, 1980; Jonides, 1981; Weichselgartner and Sperling, 1987; Müller and Rabbitt, 1989; Nakayama and MacKeben, 1989; Cheal and Lyon, 1991; Egeth and Yantis, 1997). Endogenous attention is a relatively slow process under voluntary control and its allocation to stimuli is flexible. It can be allocated to a static stimulus (fixed retinotopic location) as well as dynamic stimuli when we track for example a moving stimulus (changing retinotopic location; Pylyshyn and Storm, 1988). Exogenous attention is a relatively fast reflexive component. It has been shown that exogenous attention can also be deployed non-retinotopically according to the motion and motion-based perceptual of grouping of stimuli (Boi et al., 2011; Theeuwes et al., 2013; Gonen et al., 2014). Hence both endogenous and exogenous attention can operate in terms of retinotopic and non-retinotopic motion-grouping based coordinate systems and can control information flow from SM to STM. In Section Sequential Metacontrast: Non-retinotopic Information Storage And Processing, we discussed findings from sequential metacontrast with cueing, indicating that feature integration within a motion stream does not depend on the spatial allocation or the timing of attention. In the same study, we have also investigated a more complex stimulus where two motion streams merge to form a more complex Gestalt (Figure 9). The stimulus consisted of four motion streams, two moving rightward and two moving leftward. Two of these streams merged at a common point. When observers were asked to report the vernier offset of this common point, the outcome did depend on the allocation of attention Figure 9). The vernier offset in the attended stream dominated the outcome (Otto et al., 2010a). To summarize, nrSM has both pre-attentive and attentive components. Storage and integration of information within motion streams are pre-attentive whereas storage and integration of information across motion streams that merge (i.e., grouped into a more complex Gestalt) are flexible and depend on attention. This modulatory effect of attention on non-retinotopic integration of information may be related to the findings of Cavanagh et al. (2008) who showed that attributes of a moving stimulus which is spatio-temporally embedded in a distractor stimulus can be integrated non-retinotopically when the moving stimulus is tracked by attention. A difference between Cavanagh et al. study and ours is that in their study color and motion attributes integrated non-retinotopically whereas letter and digit shapes did not. In our study, we showed integration for vernier offsets, which would imply integration for shapes. Future studies are needed to clarify this difference.

## Paradoxes of retinotopic sensory memory revisited

Having introduced the new model, we can now compare it to the standard model containing only *r*SM and discuss what it predicts for the data that have been problematic for *r*SM.

### Anorthoscopic perception

**Figure 10:**
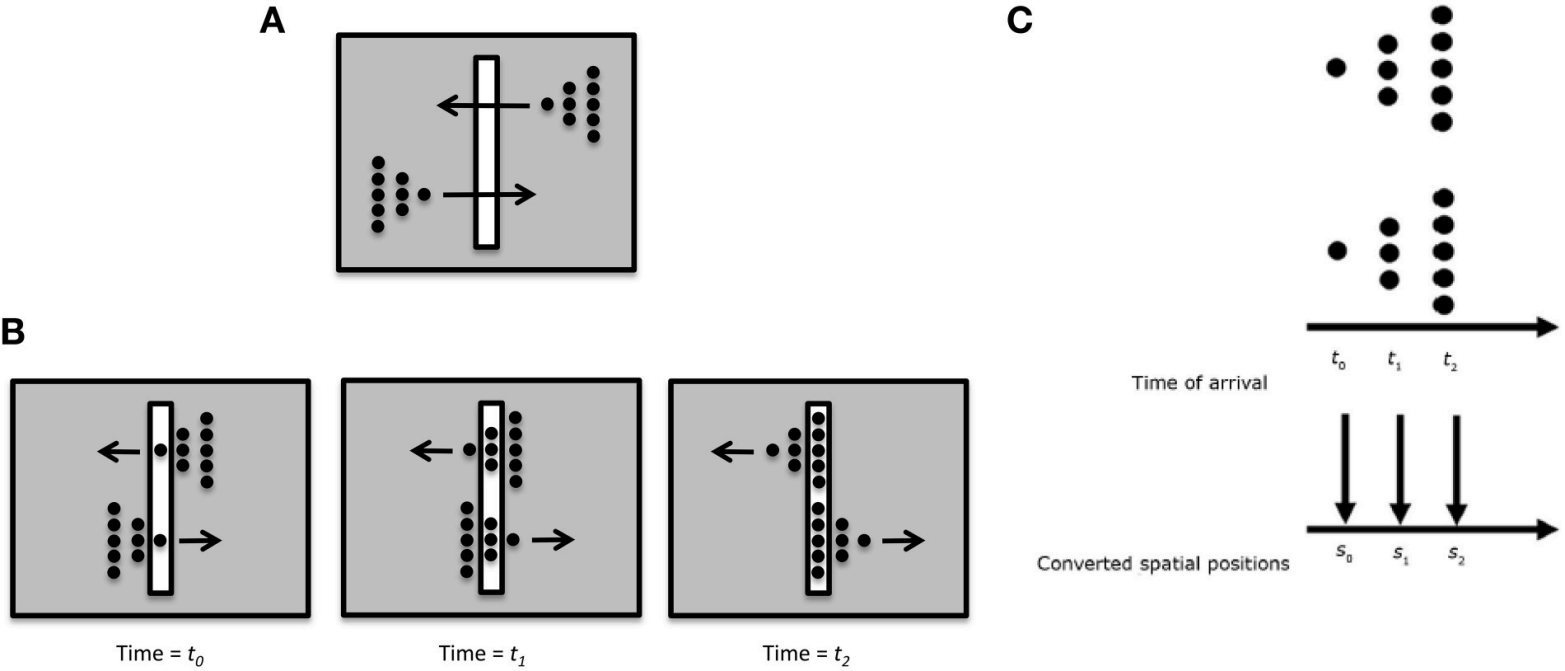
**(A)** Stimulus configuration used to test the “retinal painting” account based on *r*SM and a non-retinotopic explanation based on “time-of-arrival” coding. **(B)** Equivalent parts of the upper and lower stimuli traverse through the slit synchronously. Since an eye movement will generate the same retinal painting for the upper and lower stimuli, the retinal painting account predicts that observers will perceive the same shape for the upper and lower stimuli. **(C)** The time-of-arrival account converts the arrival times to spatial positions. Since the equivalent parts of the upper and lower stimuli ae synchronized, the time-of-arrival account also predicts that observers will perceive the same shape for the upper and lower stimuli. Contrary to these predictions, observers perceive two mirror-image triangles.

One possible way *r*SM can account for anorthoscopic perception is to assume that the observers eyes move and hence different parts of the figure fall in different parts of the retina, building up over time a retinotopic image of the stimulus which can be stored by *r*SM. In fact, this is the “retinal painting” hypothesis which was put forth by von Helmholtz (1867). While it is possible to store an anorthoscopic stimulus in *r*SM via eye movements through gradually built-up retinotopic representations, numerous studies showed that anorthoscopic perception does occur in the absence of eye movements, i.e., without retinal painting, for example by moving stimuli in opposite directions (since the eyes cannot pursue simultaneously both stimuli) or by carefully monitoring eye movements during anorthoscopic perception (McCloskey and Watkins, 1978; Rock, 1981; Morgan et al., 1982; Fujita, 1990; Sohmiya and Sohmiya, 1992, 1994; Nishida, 2004; Fendrich et al., 2005; Rieger et al., 2007). In the absence of eye movements, since the stimulus moving behind the slit activates the *same* retinotopic area successively in time, these successive stimulations should be integrated together and stored in *r*SM as a meaningless blend of different parts. To explain anorthoscopic percepts, Parks (1965) proposed a non-retinotopic memory using the “time-of-arrival coding.” The storage in this memory is based, not on retinotopic coordinates, but on *temporal coordinates* with each stimulus part assuming as its coordinate its time-of-arrival. However, time-of-arrival theory was rejected by experimental studies that used a stimulus moving to the right and its mirror-image version moving to the left (McCloskey and Watkins, 1978; Sohmiya and Sohmiya, 1992, 1994). Figure 10 shows the stimulus. Two mirror-image symmetric triangular shapes composed of dots travel in opposite directions through the slit. Their timing and speed is arranged so that equivalent parts of the upper and lower triangles pass through the slit simultaneously. If time-of-arrival were the encoding principle in non-retinotopic memory, the upper and lower stimuli should appear identical since the arrival-times of their parts are identical^4^. However, observers report, not two identical triangles, but two mirror-image symmetric triangles.

We have proposed an alternative non-retinotopic process to explain anorthoscopic percepts (Öğmen, 2007; Aydin et al., 2008, 2009;. The aforementioned experiment suggests that the critical variable is not the time-of-arrival of the stimulus but it is its direction of motion. As illustrated in Figure 7, we suggested that at a first stage motion vectors are extracted within the retinotopic slit region and these motion vectors (and not the time-of-arrival) serve as the reference-frame for *nr*SM. Accordingly, for the upper triangle, the leftward motion will be the reference-frame; whereas for the lower triangle rightward motion will be the reference frame. This allows the recovery and storage of the shape information into *nr*SM. Moreover, we made a novel prediction from our theory that shape distortions observed in anorthoscopic stimuli should be the result of differences in the perceived speeds of different parts of the stimuli. Our data provided support for this prediction (Aydin et al., 2008).

Hence, during anorthoscopic perception information is conveyed through *nr*SM, providing a solution to the paradox of anorthoscopic perception.

### How the visual system controls deleterious effects of *r*SM: motion deblurring

As mentioned before, the visible component (i.e., visible persistence) of the retinotopic sensory memory retains information for about 120 ms under normal viewing conditions (Coltheart, 1980). Based on this estimate, one would expect moving objects to appear highly smeared; however, our typical perception is relatively sharp and clear (e.g., Ramachandran et al., 1974; Burr, 1980; Hogben and Di Lollo, 1985; Castet, 1994; Bex et al., 1995; Westerink and Teunissen, 1995; Burr and Morgan, 1997; Hammett, 1997). This leads to two fundamental questions: (i) how does the visual system generate and store clear percepts if no meaningful information is conveyed by the SM stage of the modal model? and (ii) how does it avoid motion blur; i.e., how does the visual system control deleterious effects of *r*SM [Q2 in Section Sensory (Iconic) Memory]?

**Figure 11:**
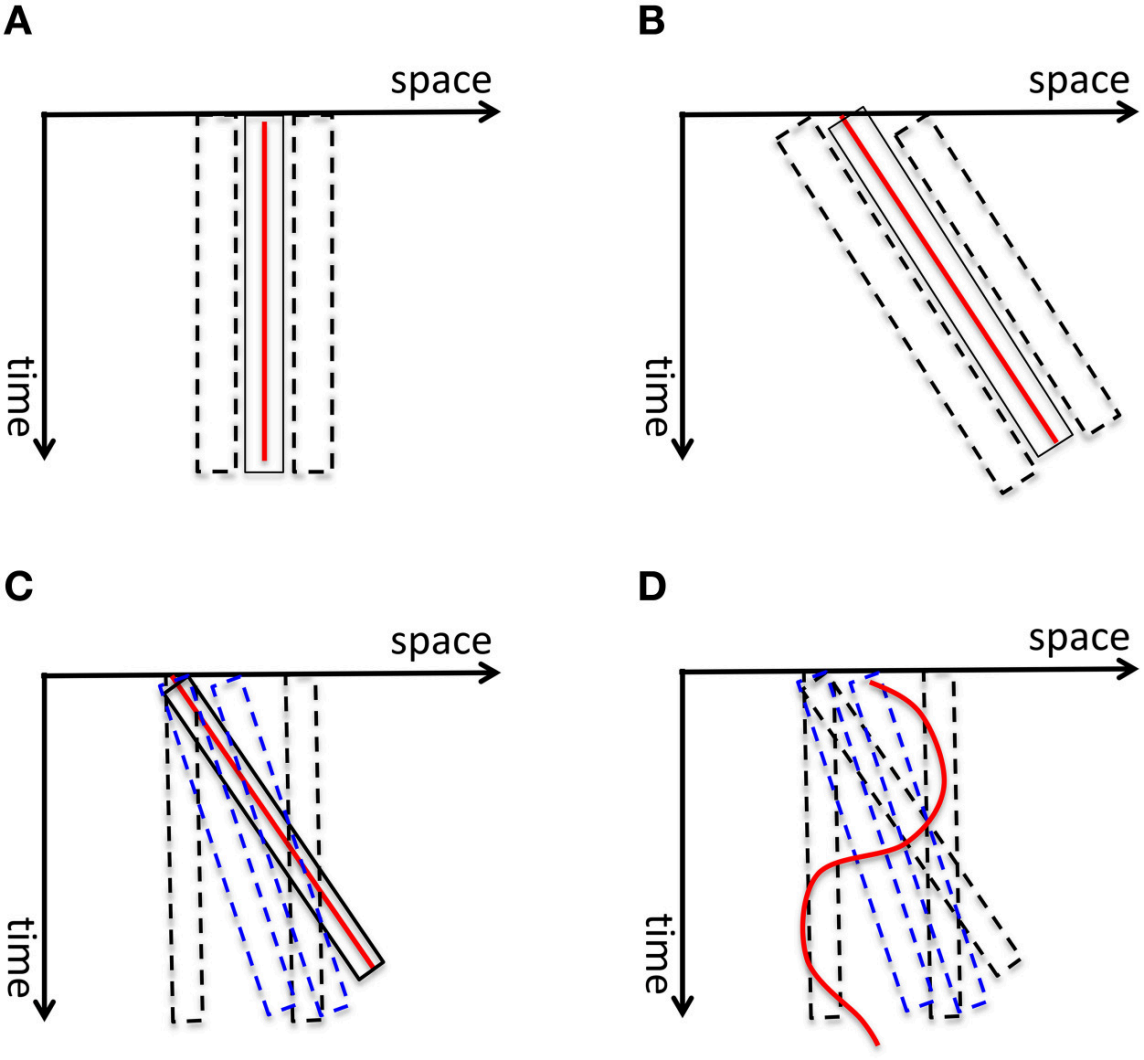
**(A)** The red vertical line depicts a static stimulus. It activates a receptive field, depicted by the solid rectangle, positioned at the location of the stimulus. The neighboring receptive fields (depicted by dashed rectangles) are not activated. Hence the activity generated by the static stimulus is spatially localized, i.e., without blur. **(B)** A stimulus moving with a constant speed is depicted by the red line in the space-time diagram. According to Burr and colleagues' theory of motion deblurring, a mechanism which is tuned to the speed of the stimulus, depicted by the solid rectangle, will integrate the stimulus along its motion path and will not generate motion blur. This is because, as in the static case in **(A),** the neighboring receptive fields are not activated. **(C)** However, other mechanisms, such as static mechanisms depicted by dashed black rectangles and motion mechanisms whose speed tuning is different than the speed of the stimulus (depicted by blue dashed lines), will be activated by the stimulus only partially and will generate residual activity that persists at their retinotopic location, hence motion blur. Bur and colleagues theory does not account how this motion blur is avoided. **(D)** Moreover, for a stimulus that moves with a more complex motion trajectory, no single mechanism will be able to integrate its information along its motion path, unless motion detectors for all possible motion paths are pre-wired at all possible retinotopic locations.

Burr and colleagues measured the perceived extent of motion blur produced by a field of moving dots and showed that it increases as a function of exposure duration up to 40 ms after which it decreases, a phenomenon called *motion deblurring* (Burr, 1980; Burr and Morgan, 1997). They proposed that spatiotemporally-oriented receptive-fields of motion mechanisms can account for motion deblurring since these receptive fields can collect information along the motion path of the object (Burr and Morgan, 1997). Hence, according to this theory, the computation of form for moving objects is carried out by motion mechanisms. To clarify this concept, consider first the space-time diagram shown in Figure 11A. The red line represents a static stimulus (since its position with respect to the horizontal space-axis is fixed). It will activate a receptive field collecting information from this position over time (depicted by the solid rectangle). Neighboring receptive fields (depicted by dashed rectangles) will not be activated since the stimulus does not fall within their “space-time window.” Hence, the activity generated by the static stimulus will be spatially localized without any blur. A stimulus moving with a constant speed can be represented by an oriented line in the space-time diagram (the red line in Figure 11B). Motion-sensitive neurons can be described by spatio-temporally oriented receptive fields (Adelson and Bergen, 1985). In terms of motion mechanisms that are tuned to the velocity of the stimulus, only one motion mechanism will be activated. Since the case in Figure 11B is equivalent to the static case in Figure 11A with a rotation, Burr and colleagues argued that the stimulus will not generate motion blur provided that it remains with the receptive field of the matching motion detector (the solid rectangle in Figure 11B) to sufficiently activate it. However, this theory fails to explain the following: As depicted in Figure 11C, mechanisms whose receptive fields are not aligned with the motion path of the object (e.g., motion detectors tuned to different speeds than the speed of the moving object; mechanisms that are not tuned to motion) will be partially activated by the moving object and will generate extensive blur. This theory cannot explain how this blur is avoided by the visual system. Furthermore, since object trajectories can be arbitrarily complex, a fixed set of oriented receptive fields cannot guarantee that a match between object motion and receptive field profile would occur in general (Figure 11D).

Kahneman et al. (1992) proposed the object-file theory to explain how the attributes of moving objects can be computed. According to this theory, an “object-file” is opened and the attributes of the moving object are inserted into this file. Since this insertion can take place over multiple retinotopic locations over the motion path, the theory could in principle provide an answer to question Q1. However, the theory gives no details, nor mechanisms to explain how object files are opened and information is inserted over the motion pathway. The theory does not answer questions Q2 and Q3 either.

Contrary to the predictions of these two theories, it has been long known that isolated targets in motion do exhibit extensive blur (Bidwell, 1899; McDougall, 1904; Dixon and Hammond, 1972; Farrell, 1984; Di Lollo and Hogben, 1985; Farrell et al., 1990). In order to reconcile the apparently contradictory observations of motion deblurring for a field of moving dots and extensive blur for isolated moving targets, we conducted experiments where we showed that (data, modeling, and review: Chen et al., 1995; Purushothaman et al., 1998; Öğmen, 2007): (1) isolated targets moving on a uniform background are perceived with extensive motion blur; (2) the presence of spatio- temporally proximal stimuli can reduce the spatial extent of perceived motion blur (motion deblurring); (3) motion mechanisms cannot account for motion deblurring; (4) metacontrast mechanisms can account for motion deblurring. Hence, to put these results in the context of our model in Figure 6, when isolated targets are in motion *r*SM becomes active and its side-effect, motion blur, is perceived. On the other hand, in the presence of spatiotemporally proximal stimuli, masking “turns off” *r*SM and no motion blur is perceived. Thus, the answer to the question Q2 is: visual masking. While earlier analyses also acknowledged that visual masking can turn *r*SM off under most ecologically valid viewing conditions, this observation led to a paradox: If the contents of *r*SM are suppressed during natural viewing, no information can be conveyed to WM and LTM, making the whole memory system inoperational under normal viewing conditions! Our theory offers a solution to this paradox by suggesting that information is conveyed to STM/WM and LTM through *nr*SM.

### What purpose does *r*SM serve?

As mentioned in the previous section, data showing that isolated moving-targets do generate motion blur indicate that *r*SM cannot be completely removed from SM, but its deleterious effects for dynamic viewing conditions are in general controlled by visual masking mechanisms. This leads to a more general question: If *r*SM cannot be eliminated from SM, is it a simple side-effect or does it serve a purpose? Ecological viewing consists of periods of fixations, saccades, pursuit, and vergence eye movements. The head and the body of the observer can be also in movement and vestibulo-ocular movements can compensate for some but not all retinotopic motions generated by these movements. For example, when the observer moves, the eyes may reflexively compensate for these movements to keep their positions on the fixation point of interest, thereby stabilizing the fixation point. However, observer's movements can also generate motion parallax for the rest of the scene and the amount of motion for different parts of the stimulus depends on the depth of objects relative to the observer. Hence, under normal viewing conditions, the retinotopic stimulus typically contains both static and dynamic components. As we have noted earlier (Footnote 2), static stimuli have a null velocity vector and their reference-frame is equivalent to a retinotopic reference frame. From a mechanistic point of view, if *nr*SM uses the activities of motion detectors to synthesize its reference-frame, in the case of static stimuli, there will be no motion-detector activity to generate the reference-frame. Hence to store information about static stimuli, a memory component that is directly anchored in retinotopic coordinates is needed and this memory component is *r*SM. Within *nr*SM, there can be multiple reference-frames deployed at different parts of the visual field depending on the motion patterns across the visual field. Hence, our theory suggests that sensory memory operates according to retinotopic motion patterns and *r*SM is a special case with a reference–frame corresponding to the null velocity. From this perspective, information can flow simultaneously from *r*SM and *nr*SM, the former carrying out the information about fixated stimuli and the rest of the scene which is static with respect to the fixated stimuli, whereas *nr*SM conveys information about objects that are in relative motion with respect to the fixation target.

## Discussion

Sensory memory was discovered in 1960s and, by the end of the decade, it became an important and integral part of the modal model of human memory. However, about two decades after its discovery, Haber placed it on a death-bed and suggested that the concept should be removed from textbooks (Haber, 1983). The inability of the sensory memory to operate under normal viewing conditions not only challenged any role it may have in information processing, but also positioned it as a “road block” to information flow from external inputs to the rest of the modal model. However, during the last decade, evidence has been accumulating on non-retinotopic processing for various stimulus attributes such as form (Nishida, 2004; Öğmen et al., 2006; Otto et al., 2006; Öğmen and Herzog, 2010), luminance (Shimozaki et al., 1999), color (Nishida et al., 2007), size (Kawabe, 2008), and motion (Boi et al., 2009; Noory et al., 2015a). We suggest that this non-retinotopic processing extends to sensory memory in the form of non-retinotopic sensory memory (*nr*SM). Furthermore, we have also shown that attention, a key process in the transfer of information from SM to STM, also operates on motion-based non-retinotopic coordinates (Boi et al., 2009, 2011). Based on these findings, we proposed here a new model for SM and discussed how it can resolve the paradoxes that stem from the Achilles' heel of the traditional SM, namely its retinotopic basis.

The traditional SM has been conceptualized as a low-level, image-like representation. However, our results and model suggest that grouping operations already take place in SM. One can also trace the roots of processing stages, such as object permanence and invariance, hitherto thought to take place at higher levels, already in SM. Having a flexible motion-based reference-frame makes this memory position-invariant. Moreover, the ability to carry information across occlusions plays a key role in achieving object permanence. Having these properties already at the SM level does make sense if one considers the ecology of vision. Gestalt psychologists have long argued that atomistic approaches, which build complex percepts by gradually combining simpler ones, cannot handle the complexity of our visual environment and grouping operations need to take place early on. Gibsonian ecological optics (Gibson, 1979) emphasizes the importance of motion in a natural environment. Duncker's (1929) and Johansson's (1975) work provided several examples of relativity of motion and the underlying motion-based reference frames (reviews: Mack, 1986; Öğmen and Herzog, 2015). Our new model for sensory memory combines these concepts and suggests how memory systems can be interfaced to our natural environment.

## Author contributions

HO, Developed the theory; wrote the original manuscript draft. MH, Developed the theory; read and commented on the original manuscript draft.

### Conflict of interest statement

The authors declare that the research was conducted in the absence of any commercial or financial relationships that could be construed as a potential conflict of interest.

## Footnotes

^1^More recently, a new type of STM, called fragile STM has been proposed (Sligte et al., 2008; Pinto et al., 2013). It has been suggested that fragile STM takes an intermediate position between SM and STM. Whether this is a memory component genuinely distinct from STM is under debate (e.g., Matsukura and Hollingworth, 2011; Makovski, 2012). In general, fragile STM seems to share the properties of STM, rather than SM. However, because it has not been tested with moving stimuli, it is difficult to compare it with the non-retinotopic sensory memory that we discuss here. We can draw, however, two important distinctions, between non-retinotopic memory that we discuss and fragile STM: Fragile STM is very sensitive to attention and cueing; but as we discuss in Sections Sequential Metacontrast: Non-Retinotopic Information Storage and Processing and A New Conceptualization of Human Sensory Memory, the role of attention in non-retinotopic memory depends on the stimulus configuration. Second, interference (or masking) happens to the contents of fragile STM when similar stimuli are presented at the same locations. As we discuss in Section Sequential Metacontrast: Non-retinotopic Information Storage and Processing, even though stimulus can be completely masked, its informational contents are robust in non-retinotopic memory.

^2^A distinction has been made between visible persistence vs. informational persistence (Coltheart, 1980). Some authors use the term iconic memory only for the informational persistence component of visual SM, whereas others use for both visual and informational components. We use the terms Sensory Memory and iconic memory to include both visible and informational persistence components.

^3^Note that for static stimuli and static observer, the velocity is 0 and hence “motion-based” reference-frame with a null velocity vector becomes identical to a retinotopic reference-frame. However, from a mechanistic point of view, if *nr*SM uses the activities of motion detectors to synthesize its reference-frame, in the case of static stimuli, there will be no motion-detector activity to generate the reference-frame. Hence to store information about static stimuli, a memory component which is directly anchored in retinotopic coordinates (*r*SM) is needed. This is analogous to on and off channels in the visual system. Although these channels can be viewed as part of a single continuum of contrast, computationally, they involve different operations and are represented by separate distinct channels.

^4^Note that there is a slight difference in the way the individual disks arrive within the slit; according to time-of-arrival coding, the disks of the top triangle will be constructed from left to right whereas the disks of the bottom triangle will be constructed from right to left. However, in both cases, the same global triangular shape will emerge from these disks regardless whether the individual disks are constructed from left to right or right to left.
